# Safety and Efficacy of SHURUI Single-Port Serpentine-Arm Robotic Surgery System in Lung Surgery: A Prospective Single-Centre Study

**DOI:** 10.1093/icvts/ivaf232

**Published:** 2025-10-31

**Authors:** Chang Li, Zhuolin Xie, Cheng Ding, Jun Chen, Xinyu Zhu, Yifan Shi, Ziyao Fang, Diego Gonzalez-Rivas, Jun Zhao

**Affiliations:** Department of Thoracic Surgery, The First Affiliated Hospital of Soochow University, Suzhou 215000, China; Institute of Thoracic Surgery, The First Affiliated Hospital of Soochow University, Suzhou 215000, China; Department of Thoracic Surgery, The First Affiliated Hospital of Soochow University, Suzhou 215000, China; Institute of Thoracic Surgery, The First Affiliated Hospital of Soochow University, Suzhou 215000, China; Department of Thoracic Surgery, The First Affiliated Hospital of Soochow University, Suzhou 215000, China; Institute of Thoracic Surgery, The First Affiliated Hospital of Soochow University, Suzhou 215000, China; Department of Thoracic Surgery, The First Affiliated Hospital of Soochow University, Suzhou 215000, China; Institute of Thoracic Surgery, The First Affiliated Hospital of Soochow University, Suzhou 215000, China; Department of Thoracic Surgery, The First Affiliated Hospital of Soochow University, Suzhou 215000, China; Institute of Thoracic Surgery, The First Affiliated Hospital of Soochow University, Suzhou 215000, China; Department of Thoracic Surgery, The First Affiliated Hospital of Soochow University, Suzhou 215000, China; Institute of Thoracic Surgery, The First Affiliated Hospital of Soochow University, Suzhou 215000, China; Department of Thoracic Surgery, The First Affiliated Hospital of Soochow University, Suzhou 215000, China; Institute of Thoracic Surgery, The First Affiliated Hospital of Soochow University, Suzhou 215000, China; Department of Thoracic Surgery, The First Affiliated Hospital of Soochow University, Suzhou 215000, China; Department of Thoracic Surgery and Minimally Invasive Thoracic Surgery Unit (UCTMI), Coruña University Hospital, Coruña 15006, Spain; Department of Thoracic Surgery, The First Affiliated Hospital of Soochow University, Suzhou 215000, China; Institute of Thoracic Surgery, The First Affiliated Hospital of Soochow University, Suzhou 215000, China

**Keywords:** single-port robotic surgery, anatomical lung resection, safety, efficacy

## Abstract

**Objectives:**

The integration of robot-assisted thoracic surgery (RATS) and uniportal video-assisted thoracic surgery (VATS) is poised to become a key direction for future advancements. The SHURUI single-port (SP) Robotic Surgery System, China’s first domestically developed SP endoscopic system, has shown promise in urological and gynaecological surgeries but lacks thoracic application data. This study evaluates its safety and efficacy in anatomical lung resection.

**Methods:**

A prospective single-centre trial enrolled 15 patients undergoing robotic pulmonary resection. The SHURUI system, featuring serpentine-arm instruments with dual continuum mechanisms, was deployed via a fifth/sixth intercostal incision. Primary endpoints included the non-conversion rate and the incidence of device-related or potentially device-related surgical complications meeting Clavien-Dindo grade 3 or higher criteria; secondary endpoints encompassed operative time, blood loss, pain scores, surgeon satisfaction, the incidence of adverse events, transfusion rates, 30-day readmission rates, 30-day reoperation rates, and 30-day mortality.

**Results:**

All procedures (8 lobectomies, 5 segmentectomies, and 2 sleeve lobectomies) achieved 100% non-conversion, with one case requiring an auxiliary port. Median total operative time, port creation time, docking time, and console time were 174 minutes (117-335), 14 minutes (8-30), 4 minutes (3-6), and 117 minutes (56-233), respectively. Blood loss was 100 mL (10-300), and the median hospital stay was 5 days (3-12). Postoperative pain scores according to the numerical rating scale (NRS) were 3, 3, and 2 at 1 hour, 24 hours, and 72 hours. Surgeons reported 95/100 median satisfaction. Two minor complications (prolonged air leak, anaemia) resolved conservatively; one readmission for pleural effusion required thoracentesis. No mortality was observed within the 30-day postoperative period.

**Conclusions:**

This study demonstrates the feasibility and acceptable safety of using the SHURUI SP robotic surgery system for anatomical lung resection.

**Clinical registration number:**

The study protocol was registered at http://www.chictr.org.cn/ (ChiCTR2400084046).

## Graphical Abstract

**Figure:**
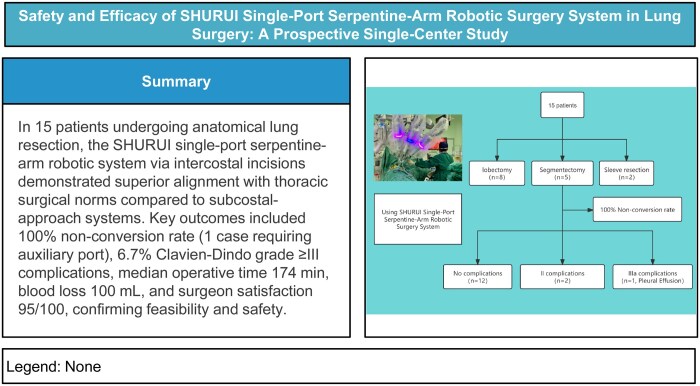

## INTRODUCTION

Robot-assisted endoscopic surgery systems represent a cutting-edge integration of multidisciplinary technologies, encompassing clinical medicine, biomechanics, mechanical engineering, computer science, and microelectronics. The core design philosophy is to leverage robotic and automated methodologies to perform complex surgical procedures with high precision under minimally invasive conditions.^1^^,^^2^

Minimally invasive pulmonary resection has become a primary surgical approach for lung cancer treatment. In 2002, Melfi et al. reported the first case of robot-assisted thoracic surgery (RATS) for pulmonary resection.^3^ Uniportal video-assisted thoracic surgery (VATS), which involves performing intrathoracic procedures through a single small incision, has gained popularity due to its superior cosmetic outcomes and improved postoperative pain satisfaction. The integration of RATS and uniportal VATS is poised to become a key direction for future advancements.

The da Vinci single-port (SP) robotic system (Intuitive Surgical, United States) was the first SP robotic-assisted surgical platform to be applied in clinical practice. It employs a cable-driven mechanism with pulley systems, enhancing manoeuvrability by incorporating an elbow joint in addition to wrist-like articulation, thereby expanding the operative workspace and reducing instrument arm collisions.^4^ However, it still presents certain limitations and challenges when applied across different surgical specialties. The SHURUI SP robotic system (Beijing, China) represents China’s first domestically developed SP endoscopic surgical system.^5^ Having received approval from the National Medical Products Administration (NMPA), it has garnered significant attention among surgeons. Preliminary successful applications have been reported in urological and gynaecological surgeries,^6–8^ but data on its use in thoracic surgery remain lacking. This study aims to evaluate the efficacy and safety of this SP robotic system in lung surgical procedures. This manuscript is written following the STROCSS reporting checklist (**Supplementary material**).

## MATERIALS AND METHODS

### Study design

This trial adopted a prospective, single-centre, single-arm design, conducted in full compliance with the ethical principles outlined in the Declaration of Helsinki. The target enrolment was set at 15 participants. The study protocol was registered at http://www.chictr.org.cn/ (ChiCTR2400084046). This study was approved by the Ethics Committee of The First Affiliated Hospital of Soochow University (No. 2024117), and written informed consent was obtained from all patients. The inclusion and exclusion criteria are presented in **Table 1**.

**Table 1.** Inclusion and Exclusion Criteria

Inclusion criteria	Exclusion criteria
Aged ≥18 years, regardless of sex; Required endoscopic surgical intervention with an indication for pulmonary segmentectomy/lobectomy; Preoperative American Society of Anesthesiologists Physical Status Classification of I-III; Voluntarily consented to participate in the trial, with written informed consent obtained from either the participant or their legal guardian; Willing to comply with follow-up visits and required examinations	History of other malignancies, as deemed ineligible by the investigator; Severe comorbidities (eg, cardiac, pulmonary, hepatic, cerebral, or renal dysfunction) or general frailty rendering them unfit for general anaesthesia or surgery; Significant bleeding tendency or coagulopathy; Active infectious diseases or other severe non-communicable infections; HIV-positive or *Treponema pallidum* (syphilis)-positive status; Severe allergic predisposition, or suspected/confirmed alcohol, drug, or substance abuse; History of epilepsy, psychiatric disorders, or cognitive impairment; Pregnancy, lactation, or planned pregnancy during the trial period (for female participants); Participation in other interventional clinical trials within 3 months prior to signing informed consent; Any other condition considered unsuitable by the investigator.

### Surgical platform

The SHURUI SP surgical robotic system consists of a surgeon console, patient-side surgical platform, 3-dimensional electronic endoscope, and specialized surgical instruments with accessories. This innovative system incorporates serpentine surgical instruments featuring “dual continuum mechanism” technology, allowing both the instruments and endoscope to be introduced into the body cavity through a specially designed flat triple-channel cannula.^5^ Unlike multi-port systems with 4 arms, the SHURUI SP system employs 2 instrument arms and 1 endoscope integrated into the cannula. This configuration reduces the thoracic occupancy and collision risks, while assistant access gap enables dynamic tissue retraction without requiring additional ports. During procedures, the external positioning arms remain stationary, effectively eliminating the risk of instrument collision. The highly articulated serpentine arms provide exceptional intraoperative manoeuvrability, significantly expanding the operative workspace while maintaining optimal spatial orientation. When combined with the endoscope, this configuration offers surgeons both an expansive surgical field and superior operational angles for precise intervention.

### Perioperative procedure

Upon hospital admission, all patients underwent comprehensive medical history documentation, laboratory testing, and imaging examinations to verify eligibility based on the predefined inclusion/exclusion criteria. Following confirmation of surgical feasibility, informed consent was obtained, and preoperative respiratory preparation was initiated.

**Figure 1.**
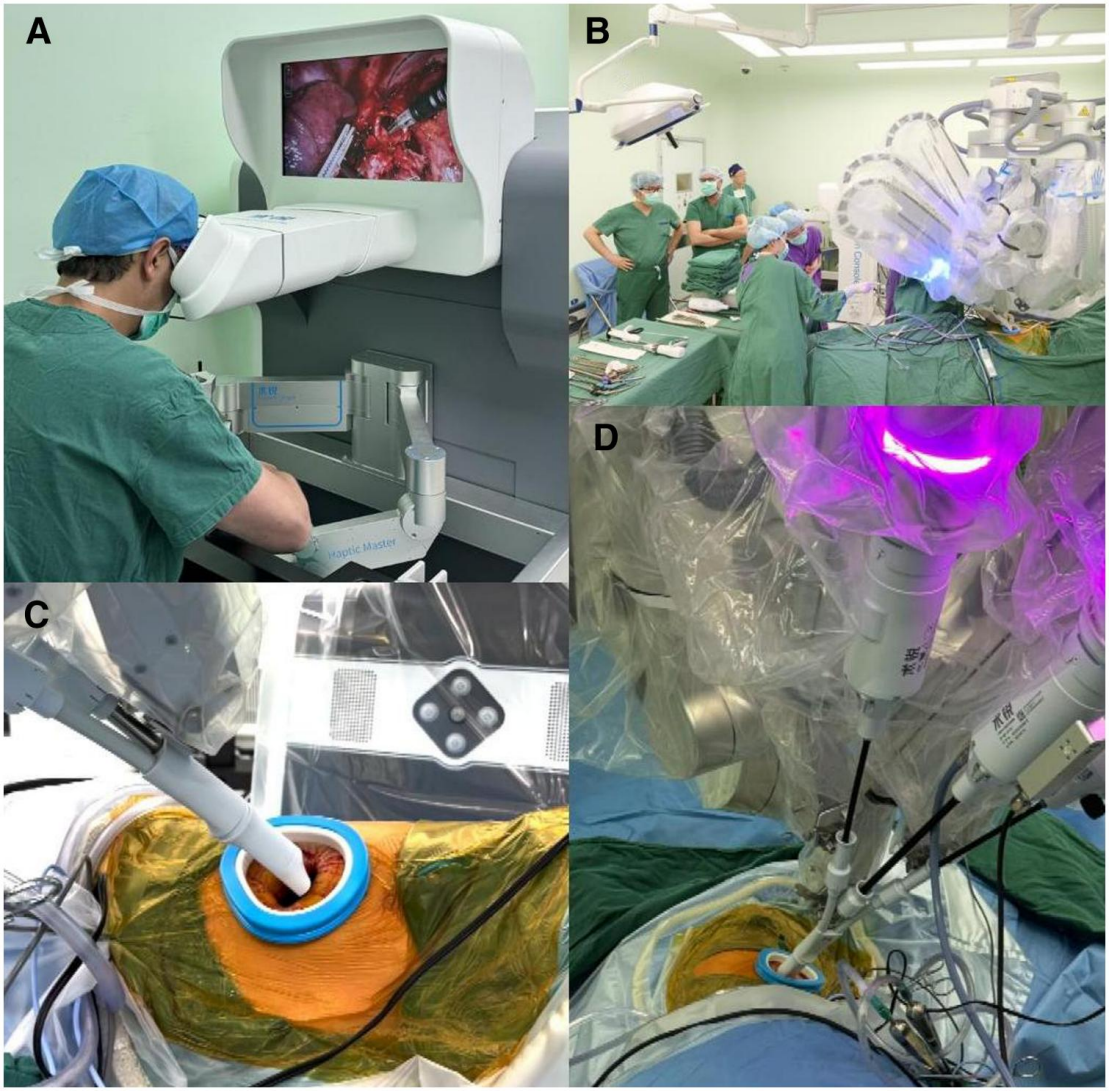
Components of the SHURUI SP Surgical Robotic System. (A) Surgeon manipulates robotic arms through a high-definition interface at the console. (B) External view of the patient-side surgical platform. (C) Customized flat cannula accessing the thoracic cavity via intercostal incision. (D) Single-arm deployment of instruments and endoscope through the cannula, with external robotic arm maintaining positional stability. Abbreviation: SP, single port

**Figure 2.**
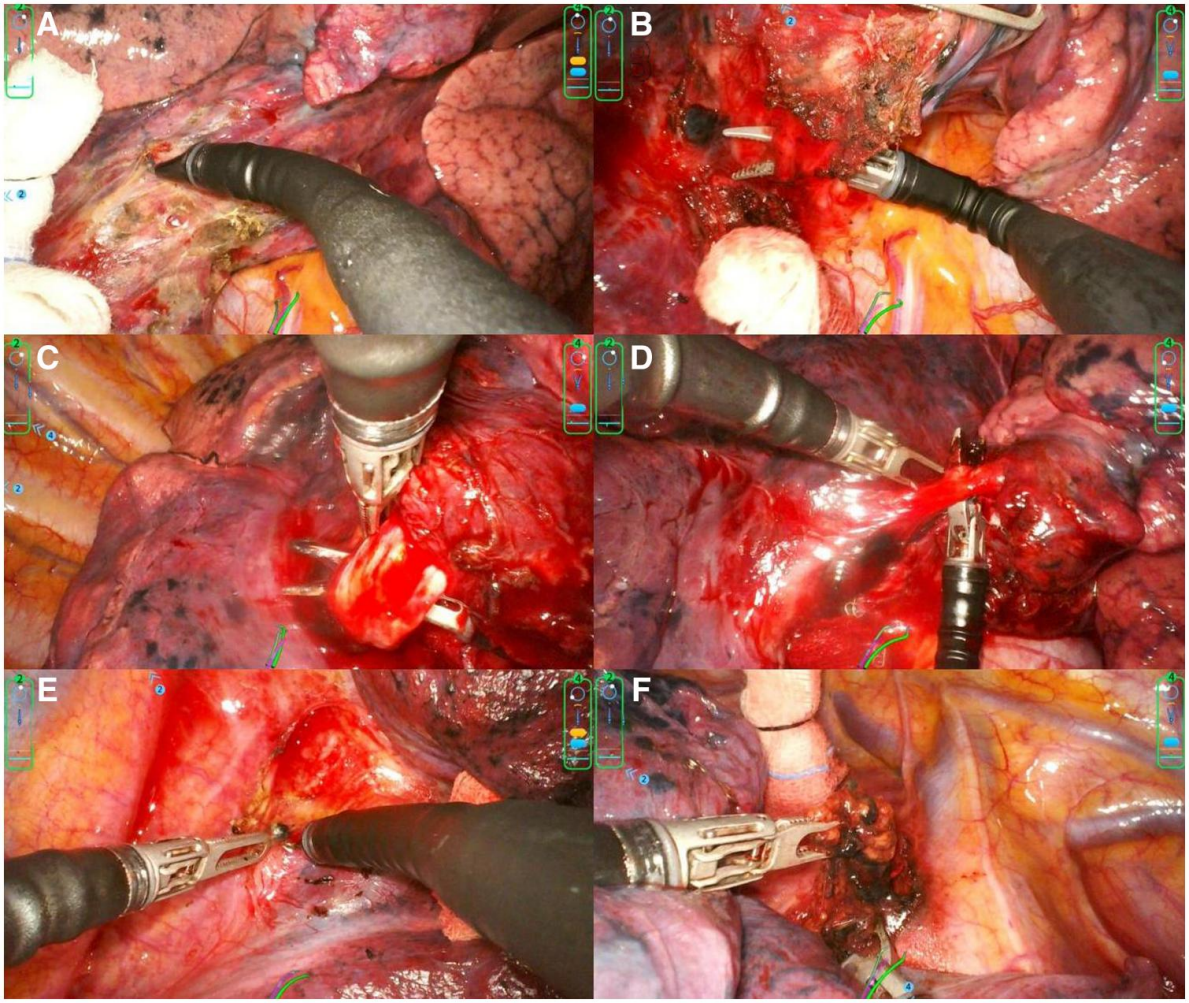
Operative Workflow of the SHURUI SP Surgical Robotic System in Standard Anatomical Lung Resection (Exemplified by Right Middle Lobectomy). (A) Dissecting the underdeveloped interlobar fissure. (B) Isolating the right middle pulmonary vein. (C) Dissecting the right middle bronchus. (D) Isolating the right middle pulmonary artery. (E) Lymph node dissection at the pulmonary hilum and peribronchial region. (F) Mediastinal lymph node dissection. Abbreviation: SP, single port

**Figure 3.**
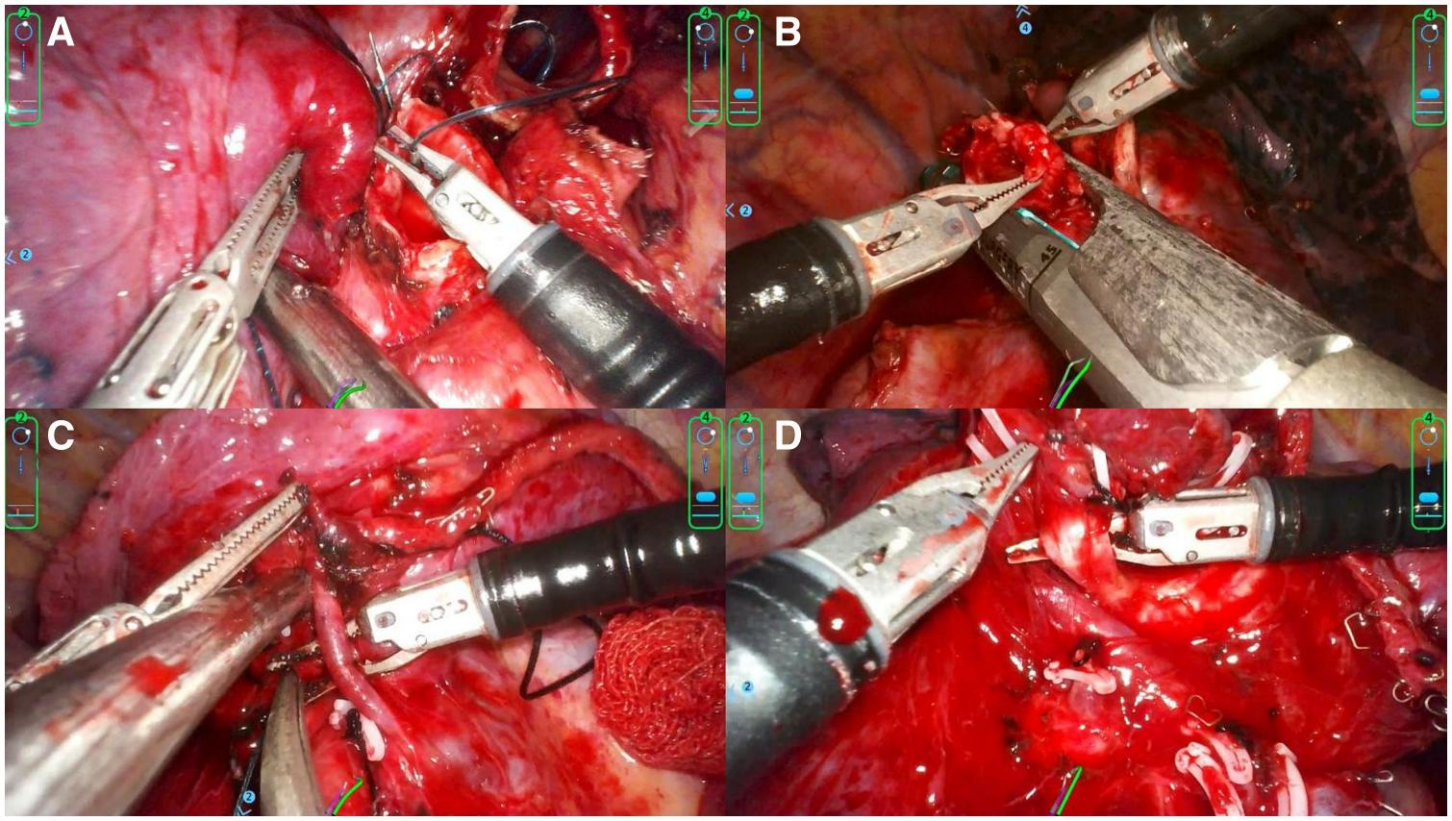
Key Procedural Steps of the SHURUI SP Surgical Robotic System in Complex Pulmonary Surgery. (A) Bronchial anastomosis using PROLINE suture. (B) Bronchoplasty following bronchial transection. (C) Dissection and mobilization of subsegmental pulmonary vessels. (D) Isolation of subsegmental bronchi. Abbreviation: SP, single port

**Figure 4.**
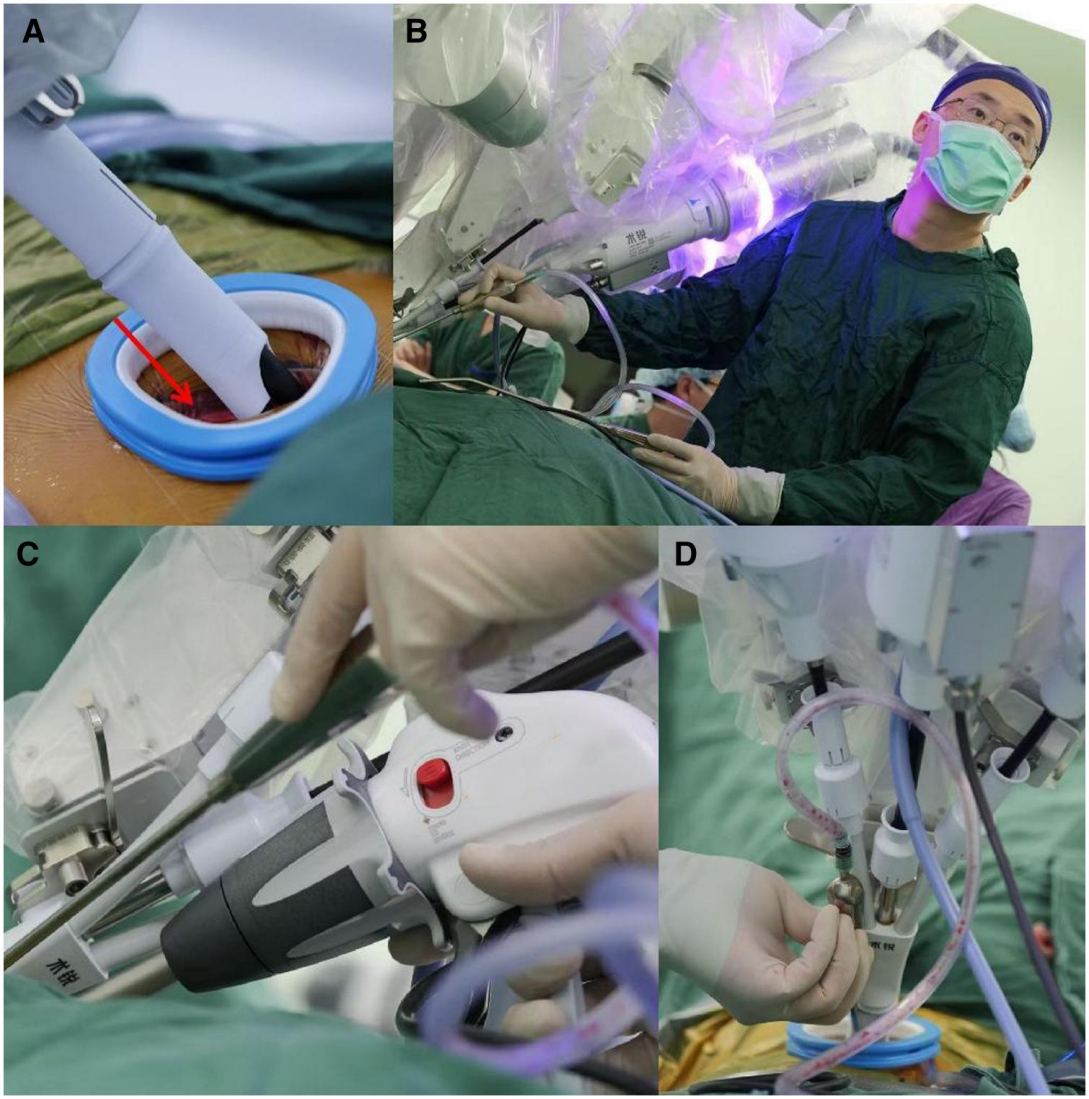
The Application of Non-Robotic Instruments: (A) Non-robotic instruments are introduced through the physical space adjacent to the flat cannula of the intercostal incision. (B) An assistant is a senior and highly experienced surgeon. (C) The use of linear cutting and closing devices. (D) The use of a specifically designed slender suction apparatus.

On the day of surgery, under general anaesthesia, patients were positioned in the lateral decubitus position. The surgical approach was selected based on resection targets: a fifth intercostal incision was typically used for upper lobectomy, while the fifth or sixth intercostal space was chosen for middle/lower lobectomy. The intercostal incision approach was identical to that of conventional uniportal VATS. After thoracic access and robotic system docking, a triple-channel flat cannula was inserted through the incision to accommodate 1 endoscope and 2 instrument arms (typically a bipolar forceps, a Maryland dissector, or a monopolar electrocautery hook) (**Figure 1**). Under high-definition endoscopic guidance, meticulous dissection was performed to isolate the arteries, veins, and bronchial structures, followed by division of the vessels/bronchus and systematic lymph node dissection (**Figure 2**). In some complex surgeries, subsegmental vessels and bronchi are meticulously managed to achieve combined subsegmentectomy, or bronchial anastomosis is performed during sleeve lobectomy (**Figure 3**). In special cases, bronchoplasty may be accomplished by incising the bronchus followed by endostapler closure. The small segmental arteries and veins were ligated using sutures or Hem‐o‐lok. Other large segmental arteries and veins, lobar arteries and veins, fused fissures, and bronchi were divided using an endostapler. Non-robotic instruments (such as Hem-o-lok clips and endoscopic staplers) were manipulated by the assistant through the physical space adjacent to the cannula within the same intercostal incision (indicated by arrow in **Figure 4A**). This was achieved without disengaging the robotic cannula, maintaining continuous SP access. The stapling is performed by the assistant (**Figure 4**). All procedures were conducted by the same experienced surgical team. The annual caseload of the team exceeds 50 robotic thoracic procedures, with >500 uniportal VATS operations per lead surgeon. The host institution of this research team has organized multiple international workshops on SP robotic surgery. Haemostasis, chest tube placement, and wound closure were performed after robotic undocking.

Postoperatively, patients received routine prophylactic antibiotics, expectorants, analgesia, and supportive care. Follow-up assessments at 1 hour, 24 hours, and 72 hours included numerical rating scale (NRS) pain scoring and complication surveillance. Chest tube removal and discharge timing were determined by clinical status, imaging findings, and therapeutic response. A 30-day outpatient review with repeat laboratory/imaging studies was conducted to evaluate complications and the need for reintervention or readmission.

### Data collection and assessment of surgical outcome

The dataset demonstrated complete integrity, with no missing values for any analysed variable across all 15 enrolled patients. Comprehensive data capture was ensured through dual-source verification of electronic medical records and synchronized robotic system logs. The primary effectiveness endpoint of this trial was the non-conversion rate of surgical procedures. Surgical conversion was defined as the need to transition from the SP endoscopic surgical system to either alternative instrument control systems, conventional video-assisted thoracoscopic surgery, or open thoracotomy. Secondary effectiveness endpoints included intraoperative blood loss, operative time, length of hospital stay, postoperative wound pain scores, and surgeon satisfaction. We meticulously documented the following time parameters: initial body cavity incision time, time of platform docking start, time of platform docking complete, commencement and conclusion of surgeon instrument manipulation, and time of surgical skin closure. Total operative time was defined as the duration from body cavity incision to skin closure. Port creation time encompassed the period from initial incision to time of platform docking start. Docking time was defined as the duration from platform docking start to complete. And console time referred specifically to the interval between the surgeon’s initiation and termination of robotic instrument control. Hospital stay duration was calculated as the total number of inpatient days from the day of surgery to discharge.

For safety evaluation, the primary endpoint was the incidence of complications meeting Clavien-Dindo grade 3 or higher criteria, occurring between the initial body cavity incision and 30 days postoperatively. Secondary safety endpoints included the transfusion rates, 30-day readmission rates, 30-day reoperation rates, and 30-day mortality. Notably, readmissions and reoperations unrelated to pulmonary surgical causes were excluded from these calculations.

### Data analysis

Statistical analysis was performed using the IBM SPSS Statistics, version 26.0 (IBM Corporation, Armonk, NY, United States). Continuous variables are reported as median (minimum, maximum). Categorical variables were presented as frequencies and percentages.

## RESULTS

The demographic and clinical characteristics of the 15 enrolled patients are summarized in **Table 2**. The cohort comprised 6 male (40%) and 9 female (60%) patients, with a median age of 61 years (27, 81). Lobectomy and adenocarcinoma represented the predominant surgical approach and pathological diagnosis, respectively. The procedures comprised 8 lobectomies (53.3%: 3 right upper, 2 right middle, 2 right lower, and 1 left upper lobe), 5 segmentectomies (33.3%: 2 left S^3^, 1 right S^1^, 1 right S^3^, and 1 right S^2b+3a^), and 2 sleeve resections (13.3%: 1 right upper and 1 right lower lobe).

**Table 2.** Characteristics of Patients and Lesions

Variable	Study cohort (*N* = 15)
Age (years)	61 (27, 81)
Body mass index (kg/m^2^)	22.1 (16.22, 27.34)
Gender, *n* (%)	
Male	6 (40%)
Female	9 (60%)
ASA physical status classification system, *n* (%)	
ASA I	6 (40%)
ASA II	9 (60%)
Lesion location, *n* (%)	
Right upper lobe	7 (46.7%)
Right middle lobe	2 (13.3%)
Right lower lobe	3 (20%)
Left upper lobe	3 (20%)
Surgical approach, *n* (%)	
Lobectomy	8 (53.3%)
Segmentectomy	5 (33.3%)
Sleeve resection	2 (13.3%)
Pathological outcome, *n* (%)	
Adenocarcinoma	8 (53.3%)
Microinvasive adenocarcinoma	3 (20%)
Squamous carcinoma	2 (13.3%)
Benign lesion	2 (13.3%)
Systematic lymph node dissection, *n* (%)	
Yes	8 (53.3%)
No	7 (46.7%)
Number of groups for lymph node dissection	3 (0, 6)
Pathological T stage, *n* (%)	
T1	10 (66.7%)
T2	3 (20%)
NA	2 (13.3%)
Pathological N stage, *n* (%)	
N0	11 (73.3%)
N1	1 (6.7%)
N2	1 (6.7%)
NA	2 (13.3%)

Abbreviation: ASA, American Society of Anesthesiologists. NA, Not Applicable.

Regarding efficacy outcomes (**Table 3**), the surgical non-conversion rate was 100%, indicating all procedures were successfully completed using the SP endoscopic surgical system without conversion to conventional thoracoscopic or open surgery. In one case, an additional auxiliary port was created to facilitate optimal placement of a vascular clamp from a specific angle. All surgical procedures achieved R0 resection (microscopically negative margins). The median total operative time, port creation time, docking time, and console time were 174 minutes (117, 335), 14 minutes (8, 30), 4 minutes (3, 6), and 117 minutes (56, 233), respectively. Median intraoperative blood loss was 100 mL (10, 300). Postoperative pain assessment revealed median NRS scores of 3 (1, 5), 3 (1, 4), and 2 (1, 3) at 1-hour, 24-hour, and 72-hour time points, respectively. The median length of hospital stay was 5 days (3, 12). Based on a 100-point satisfaction scale, surgeons reported a median satisfaction score of 95 (86, 100) upon completion of every operation.

**Table 3.** Outcomes and Results for Evaluating Efficacy and Safety

Variable	Study cohort (*N* = 15)
Non-conversion rate, *n* (%)	15 (100%)
R0 resection rate, *n* (%)	15 (100%)
Rate of additional port placement, *n* (%)	1 (6.7%)
Total operative time (minutes)	174 (117, 335)
Port creation time	14 (8-30)
Docking time (minutes)	4 (3-6)
Console time (minutes)	117 (56, 233)
Intraoperative blood loss (mL)	100 (10, 300)
Length of hospital stay (days)	5 (3, 12)
NRS pain score	
1 hour after surgery	3 (1, 5)
24 hours after surgery	3 (1, 4)
72 hours after surgery	2 (1, 3)
Surgeon satisfaction score	95 (86, 100)
Complications, *n* (%)	
No complications	12 (80%)
II	
Moderate anaemia	1 (6.7%)
Air leak	1 (6.7%)
IIIa	
Pleural effusion	1 (6.7%)
Blood transfusion rate, *n* (%)	1 (6.7%)
30-day hospital readmission rate, *n* (%)	1 (6.7%)
30-day reoperation rate, *n* (%)	0 (0)
30-day mortality rate, *n* (%)	0 (0)

Abbreviation: NRS, numerical rating scale.

Regarding safety outcomes (**Table 3**), the incidence of Clavien-Dindo grade III or higher complications was 6.7%, with no mortality observed. During postoperative hospitalization, 2 patients experienced complications: one developed prolonged air leakage that was successfully managed through negative-pressure suction and intrapleural glucose injection, ultimately achieving uneventful extubation and discharge; the other required transfusion of 2 units of modified allogeneic plasma (MAP) for moderate anaemia detected on postoperative blood tests. Additionally, one patient was readmitted within 30 days postoperatively due to moderate pleural effusion on the operative side, confirmed by outpatient chest radiography and accompanied by subjective chest tightness, which was effectively treated with therapeutic thoracentesis.

## DISCUSSION

The early exploration of SP robotic systems in thoracic surgery can be traced back to 2014, when Seong et al. pioneered the application of the da Vinci Si robotic system via a uniportal lateral thoracic approach for mediastinal tumour resection, later extending it to a subxiphoid approach. Subsequent studies further expanded its utility, yet notable limitations emerged, including restricted instrument manoeuvrability and challenges in addressing lesions proximal to the incision.^9^^,^^10^ The advent of the da Vinci SP system, integrating 4 articulating arms into a single robotic arm, marked a technological leap by enhancing operational flexibility through a single incision. In 2019, Gonzalez et al. systematically evaluated the da Vinci SP system in cadaveric models for thoracic procedures, concluding that its 2.8-cm diameter trocar rendered it suboptimal for intercostal access. Instead, they advocated subxiphoid entry for thymectomy and subcostal approaches for pulmonary resections.^11^ Building on these findings, Lee et al. demonstrated in 2022 the technical feasibility and safety of da Vinci SP-assisted subcostal pulmonary lobectomy/segmentectomy in 25 patients. However, these pioneering efforts revealed critical limitations: the subcostal approach imposed spatial constraints for complex pulmonary procedures, hindering intricate tissue manipulation.^12^ In 2021, Gonzalez et al. successfully performed SP robotic lobectomy utilizing the da Vinci Xi robotic surgical system. However, it should be noted that this platform was not originally designed for SP access, which consequently necessitated the operator’s extensive surgical expertise along with technical refinements of both the instrumentation and procedural techniques.^13^^,^^14^ These observations underscore the imperative for technological refinements to achieve broader clinical adoption. In this context, the SHURUI SP serpentine-arm robotic surgical system has effectively addressed these challenges through clinical implementation. Preliminary clinical observations suggest potential advantages in critical surgical parameters such as intraoperative blood loss minimization and enhanced postoperative functional recovery. To expand its intended clinical applications beyond the existing clinical division, this study evaluated the safety and effectiveness of this experimental system in thoracic surgery. Preliminary results demonstrate satisfactory outcomes across various evaluation parameters.

In this study, the non-conversion rate remained 100%, validating the system’s reliability for thoracic pulmonary procedures. This performance aligns with outcomes from previous da Vinci SP robotic studies, including a 25-patient cohort (100%) and a 35-patient study (97.1% conversion-free rate).^12^^,^^15^ The reported complications mirrored those documented in these benchmark trials, with the incidence of Clavien-Dindo grade ≥III complications (6.7%) demonstrating comparability to Jun Hee Lee’s findings (4%) and Chuan Cheng’s series (5.7%).^12^^,^^15^ Notably, previous studies did not report the performance of complex procedures such as sleeve resections, whereas our cohort successfully accomplished 2 sleeve lobectomies (right upper and lower lobes) and 1 bronchoplasty procedure. The right lower lobe sleeve lobectomy, a technically demanding operation rarely performed in clinical practice, particularly highlights the system’s emerging advantages in managing intricate anatomical reconstructions.^16^ These preliminary achievements suggest the SHURUI system may overcome historical limitations of SP platforms in executing advanced pulmonary resections.

Based on a comprehensive literature review and extensive hands-on experience, we have summarized the technical characteristics of different robotic platforms for anatomical lung resection (**Table 4**). In contrast to the da Vinci SP robotic system, the SHURUI robotic platform utilizes specialized trocars to access the thoracic cavity through the fifth or sixth intercostal space, deploying instruments and the endoscope via an approach universally familiar to thoracic surgeons. Our observed port creation time appears shorter than that reported in Chuan Cheng’s study, which similarly documented this metric.^15^ The intercostal approach minimizes potential injury to the diaphragm and mediastinal structures, eliminating the need for diaphragmatic suturing or reinforcement. Furthermore, the system enables direct application of endoscopic staplers without requiring additional auxiliary ports or robotic arm retraction, achieving angulation more consistent with conventional VATS. This design may reduce the cognitive adaptation required for surgeons transitioning from VATS to robotic systems, thereby shortening the learning curve while preserving the “open-view” advantages inherent to SP robotic surgery.^17^ Finally, though surgical complications remain unavoidable, the intercostal configuration allows rapid disengagement of the robotic system in cases of uncontrollable haemorrhage, facilitating immediate conversion to uniportal VATS or thoracotomy—a critical safety feature that enhances patient security during complex procedures.^14^ The absence of a dedicated retraction arm warrants discussion. The design of SHURUI transfers the role of the fourth arm to the bedside assistant via the cannula-side gap. This trade-off preserves the core advantage: ultra-compact access enabling true SP robotics through intercostal spaces.

**Table 4.** A Comparison of Technical Characteristics of Different Robotic Platforms in Anatomical Lung Resection

Features	da Vinci Xi	da Vinci SP	SHURUI SP
Surgical incision	Usually 3 to 4 incisions, but single-port operation is also possible	Single-subcostal incision	Single-intercostal incision
Stapler control	The chief surgeon can independently operate the stapler	The operation of the stapler requires the withdrawal of the mechanical arm or the addition of an auxiliary hole	The stapler is inserted through the gap beside the trocar sleeve of the intercostal incision and operated by an assistant.
Assistant’s requirements	Higher requirements are imposed when performing single-port surgery	General requirements	Higher requirements
Clinical advantages	Mature and stable, suitable for complex surgeries	Cosmetic effect is good, and postoperative pain is mild.	A more flexible field of vision and greater degrees of freedom of the mechanical arm are able to complete complex surgeries.
Learning curve prediction	Smooth and compatible with traditional laparoscopic habits	Steep, non-traditional incision approach and high requirements for spatial coordination	Moderate, similar to the VATS perspective

Abbreviations: SP, single port; VATS, video-assisted thoracic surgery.

The favourable evaluations from surgeons underscore the clinical acceptance of the SHURUI robotic system. Feedback from surgeons highlights 2 distinct technical advantages. First, the moderate longitudinal deployment range of instruments and endoscope accommodates patients with reduced thoracic volumes or skeletal deformities such as barrel chest, ensuring optimal operative accessibility. Second, the serpentine-arm instruments provide enhanced manoeuvrability, with the endoscope capable of elevating to bypass anatomical obstacles, while the large articulation angles and diamond-shaped configuration between instruments and endoscope minimize mutual instrument interference during complex manipulations. However, the system exhibits limitations: the absence of an integrated stapling system necessitates manual assistant intervention for transecting major vessels and bronchi. Although staplers can be introduced through the same intercostal incision with angulation comparable to conventional VATS, the trocar’s occupation of the limited incision space demands considerable expertise from surgical assistants. Similarly, the frequent use of suction devices for exposure further increases the technical demands on assistants during prolonged procedures. For novice surgeons, this SP robotic system presents dual implications in technical facilitation, particularly for complex procedures like sleeve resection. While the enhanced articulation of serpentine instruments significantly simplifies deep dissection (eg, posterior hilar exposure) and intricate suturing (eg, bronchial anastomosis)—enabling intuitive multiplanar manoeuvres that previously required exceptional hand-eye coordination in conventional uniportal VATS (such as reverse-needle driving)—substantial challenges persist. Crucially, the absence of haptic feedback forces complete reliance on visual cues for tension assessment, leaving less-experienced operators vulnerable to misjudging vascular integrity or anastomotic tightness. Furthermore, despite high-definition visualization, developing spatial anticipation skills remains equally critical. Most significantly, dependence on skilled bedside assistance becomes acutely problematic in less-experienced teams; when assistant support is suboptimal, the console surgeon must simultaneously manage robotic control and direct exposure, resulting in substantially escalated cognitive load.

This study has several limitations. First, as a single-centre cohort investigation with a limited sample size, it lacks long-term follow-up data and a control group for comparative analysis. The small sample size and single-arm design inherently limit the value of causal or associative analyses between variables. Second, all procedures were performed by a homogeneous surgical team with extensive robotic expertise, where operator proficiency and case selection bias (predominantly early-stage lesions) may influence outcome generalizability. The outcomes achieved by a high-volume team at a single centre may not generalize to broader practice settings. Future multi-institutional validation with standardized protocols is warranted to evaluate system performance across varied anatomical complexities and surgeon experience levels. Subgroup analyses stratified by resection types (eg, segmentectomy vs lobectomy) and long-term oncological outcomes remain critical next steps. Additionally, formal learning curve assessments through structured training programs will be essential to determine the system’s adaptability across institutions with differing surgical infrastructures.

## CONCLUSIONS

This study demonstrates the feasibility and acceptable safety of using the SHURUI SP serpentine-arm robotic surgery system for anatomical lung resection. Larger multicentre studies are needed to validate long-term outcomes.

## Supplementary Material

ivaf232_Supplementary_Data

## Data Availability

The data underlying this article are available in the article and in its online supplementary material.
